# Factors related to the practice of vacuum-assisted birth: findings from provider interviews in Kigoma, Tanzania

**DOI:** 10.1186/s12884-021-03738-0

**Published:** 2021-04-14

**Authors:** Sunday Dominico, Mkambu Kasanga, Nguke Mwakatundu, Paul Chaote, Samantha Lobis, Patricia E. Bailey

**Affiliations:** 1 Thamini Uhai, Dar es Salaam, Tanzania; 2 President’s Office-Regional Administration and Local Government, Dodoma, Tanzania; 3 Vital Strategies, New York, USA

**Keywords:** Vacuum extraction, Vacuum delivery, Vacuum assisted birth, Emergency obstetric and newborn care, Maternal health, Training, Assisted vaginal delivery

## Abstract

**Background:**

Vacuum-assisted birth is not widely practiced in Tanzania but efforts to re-introduce the procedure suggest some success. Few studies have targeted childbirth attendants to learn how their perceptions of and training experiences with the procedure affect practice. This study explores a largely rural cohort of health providers to determine associations between recent practice of the procedure and training, individual and contextual factors.

**Methods:**

A cross-sectional knowledge, attitudes and practice survey of 297 providers was conducted in 2019 at 3 hospitals and 12 health centers that provided comprehensive emergency obstetric care. We used descriptive statistics and binary logistic regression to model the probability of having performed a vacuum extraction in the last 3 months.

**Results:**

Providers were roughly split between working in maternity units in hospitals and health centers. They included: medical doctors, assistant medical officers (14%); clinical officers (10%); nurse officers, assistant nurse officers, registered nurses (32%); and enrolled nurses (44%). Eighty percent reported either pre-service, in-service vacuum extraction training or both, but only 31% reported conducting a vacuum-assisted birth in the last 3 months. Based on 11 training and enabling factors, a positive association with recent practice was observed; the single most promising factor was hands-on solo practice during in-service training (66% of providers with this experience had conducted vacuum extraction in the last 3 months). The logistic regression model showed that providers exposed to 7–9 training modalities were 7.8 times more likely to have performed vacuum extraction than those exposed to fewer training opportunities (AOR = 7.78, 95% CI: 4.169–14.524). Providers who worked in administrative councils other than Kigoma Municipality were 2.7 times more likely to have conducted vacuum extraction than their colleagues in Kigoma Municipality (AOR = 2.67, 95% CI: 1.023–6.976). Similarly, providers posted in a health center compared to those in a hospital were twice as likely to have conducted a recent vacuum extraction (AOR = 2.11, 95% CI: 1.153–3.850), and finally, male providers were twice as likely as their female colleagues to have performed this procedure recently (AOR = 1.95, 95% CI: 1.072–3.55).

**Conclusions:**

Training and location of posting were associated with recent practice of vacuum extraction. Multiple training modalities appear to predict recent practice but hands-on experience during training may be the most critical component. We recommend a low-dose high frequency strategy to skills building with simulation and e-learning. A gender integrated approach to training may help ensure female trainees are exposed to critical training components.

**Supplementary Information:**

The online version contains supplementary material available at 10.1186/s12884-021-03738-0.

## Background

Tanzania’s maternal mortality ratio and perinatal and neonatal mortality rates have not improved over the last 10 years, and currently stand at 556 maternal deaths per 100,000 live births, 25 perinatal deaths per 1000 births, and 39 neonatal deaths per 1000 live births, respectively [1]. Most of these deaths, as well as serious morbidities, are preventable with appropriate, timely and high-quality emergency obstetric and newborn care (EmONC). The Government of Tanzania is committed to improving access to these life-saving services [2]. One component of EmONC services is assisted vaginal delivery with a vacuum extractor [3], which for some indications may be safer than cesarean delivery conducted after the start of labor [4–6]. It is useful in shortening the second stage of labor, responding to fetal distress and reducing a woman’s physical effort in the presence of other medical conditions such as cardiopulmonary compromise. Furthermore, vacuum extraction is a clinical option that can help reduce the performance of unnecessary cesarean sections [4, 6, 7].

Despite a pool of evidence and recommendations in support of vacuum extraction, the practice is underutilized and currently on a declining trend in many low- and middle-income countries including Tanzania [8]. Reasons for low provision range from a lack of functioning equipment to fears about poor neonatal outcomes [8, 9]. In settings where functional vacuum extractors are available, the practice is limited by providers’ confidence and competency, which is largely determined by providers’ work and training experiences. A global survey among public health specialists and obstetricians that measured knowledge, attitudes and training of vacuum extraction in 121 developing countries found that more than half (52%) had no or limited knowledge of the procedure [10].

Given the global recognition of the underutilization of vacuum extraction, efforts to reintroduce the procedure into several South American and sub-Saharan African countries have been undertaken (Ecuador, Mexico, Mozambique, Tanzania, Uganda) [9, 11–14]. But little is known about the longer-term retention and incorporation of the practice into routine obstetric services, or why some practitioners adopt it and others do not. In order to better understand the latter, we designed a survey of health providers to assess how different training modalities, health facility and individual characteristics affect the practice of vacuum extraction.

## Methods

### Study setting, aim and design

The cross-sectional survey design was set in the region of Kigoma, Tanzania. Kigoma has a population of 2.3 million, a population growth of 2.7% and is divided into eight administrative councils [15]. Most people in the region are subsistence farmers and reside in rural areas. In 2018, Kigoma’s institutional delivery rate was 85% (up from 49% in 2013) and the population-based cesarean delivery rate was 4.5% (up from 2.6% in 2013) [16]. In the last 3 months of 2018, only 36% of the 33 hospitals and health centers in Kigoma provided assisted vaginal delivery [17].

For over a decade, Thamini Uhai, a Tanzanian non-governmental organization, has worked closely with the government and other partners in Kigoma to develop and implement a model program to reduce maternal and neonatal mortality in remote areas. The model was developed in 2005 by the late Dr. Mbaruku (former Kigoma Regional Medical Officer), Thamini Uhai/Vital Strategies (formerly World Lung Foundation) and its partners. It was a response to the challenge of delivering good-quality EmONC to women living far from hospitals by decentralizing comprehensive EmONC services (including obstetric surgery) to the health center level. The program was implemented in 12 health centers in Kigoma. In addition, 3 existing hospitals in the region that already provided comprehensive EmONC were included and given technical support by the program to backstop health centers. Support was provided to the 15 health facilities in this study between 2006 and 2019; the most intensive period of implementation was between 2013 and 2019.

To support the delivery of good-quality comprehensive EmONC in health centers, the program constructed/renovated and equipped operating theatres and maternity wards and installed/strengthened electricity and water supply systems. Mid-level health providers, mainly assistant medical officers and nurse-midwives, were trained, mentored and supervised to perform the nine comprehensive EmONC signal functions [18]. In particular, continuous efforts were made to sustain vacuum extraction by ensuring they had functional equipment, providing regular onsite mentoring, conducting periodic skills-based continuing medical education workshops, producing an e-Learning platform for independent learning that included a module on vacuum extraction [19], providing access to senior obstetricians 24/7 via a free phone call and conducting routine clinical audits of caesarean sections and vacuum extractions. This package of clinical support was designed by the program’s implementers to enable providers working in remote parts of the region to provide comprehensive EmONC on their own. With this support, most EmONC signal functions were sustained, such as obstetric surgery and neonatal resuscitation. Yet, performance of assisted vaginal delivery across supported facilities was inconsistent over time [17].

This study aimed to interview maternal health providers about their training experiences with vacuum extraction, their knowledge of and attitudes toward vacuum extraction, and how these related to their recent performance of vacuum delivery.

### Characteristics of study facilities

Nearly half of institutional deliveries in the region are managed at the 15 selected facilities [16]. Moreover, the majority of skilled birth attendants expected to perform basic and comprehensive EmONC signal functions are located in these health facilities. Generally, the types and distribution of birth attendants posted at these facilities are similar, with nurses and medical attendants accounting for most of them, with a few assistant medical officers (AMOs) and/or medical doctors (MDs). By default, the hospitals have more staff compared to health centers.

### Participants and survey administration

The survey was conducted through face-to-face interviews with health providers and managers using a structured questionnaire. The survey questionnaire (originally in English and translated to Swahili) was developed specifically for this study by the investigators, who are all knowledgeable and experienced with the subject (*Additional file 1). To be eligible for inclusion, the provider or manager had to be present during the study visit, report having worked at their current location for at least 3 months and grant informed consent. Providers and managers were interviewed in their work-stations during the last 2 weeks of January 2019. Due to the shortage of health providers in the region, all eligible providers in health centers were invited to participate. In hospitals, only providers working in maternity units were interviewed. Managers included facility supervisors, in-charges and members of the Regional, District or Town Councils’ health management team (R/CHMT). For R/CHMT managers, the study was limited to clinicians.

Senior clinicians (AMOs/MDs) and registered nurses/midwives with experience in maternal and newborn health services administered the surveys. They received a 3-day training on the study protocol and survey instruments which included a 1-day pilot test of tools in a nearby health facility. For this study, teams of 3 deployed to the field; data collection often took 2–3 days in hospitals and 1–2 days in health centers. On average, interviews lasted between 30 and 45 min per respondent. Interviews were conducted in private confidential settings within the facility’s premises.

### Statistical analysis

Data were entered into Epi info [7] and exported to SPSS version 24 (SPSS, Chicago, IL, USA) for statistical analysis.

The primary outcome of interest was recent performance of vacuum delivery, which was self-reported. “Recent” was defined by the immediate 3-month period prior to survey. Three months was chosen to maximize recall accuracy as well as concern that less frequent clinical experience could be problematic for skill set maintenance.

The variables selected for exploration related to characteristics of the provider (sex, age, professional cadre, training experience, years since professional qualification, and knowledge and attitudes regarding vacuum extraction). Contextual variables included type of facility and administrative district. All were categorical variables.

A composite knowledge score was created for each respondent based on his or her ability to report indications and contra-indications for vacuum extraction. The questions were posed as open-ended but responses were pre-coded and required a respondent to spontaneously list all the indications and contra-indications for vacuum extraction that they could. No prompting beyond “can you think of anything else?” was done. The 10 pre-coded indications were: prolonged 2nd stage of labor, maternal exhaustion, need to shorten 2nd stage of labor for medical reasons, severe anemia, heart diseases/failure, severe pre-eclampsia or eclampsia, suspected or imminent fetal distress in 2nd stage of labor, fetal bradycardia, fetal tachycardia, and thick meconium stained liquor. The 8 pre-coded contra-indications were: breech/face/brow presentation/transverse lie, un-engaged fetal head, gestational age less than 34 weeks, incomplete cervical dilation in nulliparae, cephalo-pelvic disproportion, incomplete cervical dilation in multiparae, moulding grade 3 and HIV-infected pregnant women. Each item mentioned had the value of 1; otherwise the item was given a value of zero. Responses ranged from zero to 18 and were dichotomized into the top quartile and the lower three quartiles.

A summary index for training was calculated based on a respondent’s self-reported exposure to 11 different training opportunities. No weighting of the scores was done, thus, each reported opportunity accrued a value of 1; scores could range from 0 to 9 since a person could report only one pre-service option (with or without hands-on experience) and only one in-service option (with or without hands-on experience). This score was presented as a categorical variable: exposure to 6 or fewer training modalities and 7 or more.

Since recent performance of vacuum extraction was a dichotomous categorical variable with a yes/no answer, binary logistic regression was the most appropriate method to control for covariates. All independent variables were entered as a block in the regression. All variables were categorical. No multicollinearity among explanatory variables was found. Significance for all statistical testing was set at 95% CI (*p* < 0.05). Pearson Chi-square tests were used to test for associations between groups for all descriptive analyses.

### Ethical review and consent

The study received ethical clearance from the National Ethical Review Committee of the National Institute for Medical Research (NIMR) in Tanzania. Approval was also obtained from the Regional and District Medical Officers as well as the respective facility heads. A written informed consent was obtained for all survey participants.

## Results

In total, 297 providers were interviewed. The health providers interviewed were fairly evenly split between hospitals (54%) and health centers (46%) where they worked (Table 1). They were predominantly enrolled nurses (44%) in both hospitals and health centers. While female health workers were predominant at hospitals (64%), males were more prevalent at health centers (57%). At hospitals, 59% were under the age of 40 compared to 69% of health workers at health centers. Fifty-two percent of health workers at hospitals had graduated fewer than 8 years before this study compared to 62% among health center workers.

**Table 1:** Percent distribution of health providers according to type of facility, by select characteristics

	Total ***n*** = 297	Hospitals ***n*** = 159	Health centers ***n*** = 138	***p***-value
**Total**	100.0	53.5	46.5	
**Professional category**				
AMOs / MDs	13.5	11.3	15.9	0.016
COs	10.4	6.3	15.2	
NOs / ANOs / RNs	32.0	37.7	25.4	
Enrolled nurses	44.1	44.7	43.5	
**Sex**				
Male	45.9	36.1	57.2	< 0.001
Female	54.1	63.9	42.8	
**Age**				
21–29	41.2	34.2	49.3	0.009
30–39	22.3	24.7	19.6	
40–49	21.6	27.8	14.5	
50–60	14.9	13.3	16.7	
**Years since qualification**				
< 4 years	26.7	18.3	37.1	0.006
5–7 years	29.6	33.3	25.0	
8–14 years	20.6	22.9	17.7	
15–37 years	23.1	25.5	20.2	
**Pre- and in-service training in VE**				
Pre-service only	21.2	25.8	15.9	0.001
In-service only	14.8	5.1	9.8	
Both pre- and in-service	42.4	47.8	36.2	
Unspecified training	1.3	0.0	2.9	
None	20.2	17.0	23.9	
**Knowledge score**				
Lower 74%	74.7	79.9	68.8	0.029
Top 25%	25.3	20.1	31.2	
**Attitudinal score**				
Perceived more barriers	74.1	78.6	68.8	0.055
Perceived fewer barriers	25.9	21.4	31.2	
**Administrative Council**				
Kigoma Municipality	19.2	29.6	7.2	< 0.001
Other councils	80.8	70.4	92.9	
**Performed VE last 3 months**				
Yes	30.6	21.4	41.3	< 0.001
No	69.4	78.6	58.7	

*AMOs* assistant medical officers, *MDs* medical doctors, *COs* clinical officers, *NOs* nurse officers, *ANOs* assistant nurse officers, *RNs* registered nurses, *VE* vacuum extraction

Of the 297 providers, 60 (20%) reported no formal training in vacuum extraction (nor had they ever conducted a vacuum delivery). However, 42% reported exposure to both pre-service and in-service training in vacuum extraction. The proportion with both pre-service and in-service training was higher among hospital providers (48%) than health center providers (36%). One percent reported no formal training but because they had conducted vacuum extractions, presumably taught by their colleagues, they were considered to have had “unspecified training.” A larger proportion of health workers in health centers (31%) compared to hospitals workers (20%) scored in the top 25% of the composite knowledge index based on indications and contra-indications for vacuum extraction. Similarly, health center providers perceived fewer barriers to vacuum extraction (31%) than did hospital providers (21%). Most providers (81%) worked in administrative councils other than Kigoma Municipality, the capital of Kigoma region; this was particularly true of health center providers (93%). A larger proportion of providers who recently performed a vacuum delivery were found at health centers (41%) than at hospitals (21%).

In Table 2 we looked again at the characteristics from Table 1 to determine if they were associated with the recent performance of vacuum extraction, i.e. in the last 3 months. The characteristics associated with recent performance were being male (38% vs 25%), ever having been trained in vacuum extraction, a high knowledge score (45% vs 26%), and working in a district other than Kigoma Municipality (35% vs 12%).

**Table 2:** Percentage of providers who conducted vacuum extraction in the last 3 months, by select characteristics

	Performance in last 3 months		
	n	%	***p***-value
**Professional category**			
AMOs / MDs	40	40.0	0.130
COs	31	19.4	
NOs / ANOs / RNs	95	25.3	
Enrolled nurses	131	34.4	
**Sex**			
Male	136	37.5	0.020
Female	160	25.0	
**Age**			
21–29	122	27.0	0.692
30–39	66	34.8	
40–49	64	32.8	
50–60	44	31.8	
**Years since qualification**			
< 4 years	74	28.4	0.921
5–7 years	82	30.5	
8–14 years	57	33.3	
15–37 years	64	32.8	
**Pre- and in-service training in VE**			
Pre-service only	63	12.7	< 0.001
In-service only	44	61.4	
Both pre- and in-service	126	57.1	
Unspecified training	4	100.0	
No training	60	0	
**Knowledge score**			
Lower 75%	222	25.7	0.001
Top 25%	75	45.3	
**Attitudinal score**			
Perceived more barriers	220	29.1	0.136
Perceived fewer barriers	77	35.1	
**Administrative Council**			
Kigoma Municipality	57	12.3	0.001
Other councils	240	35.0	

*AMOs* assistant medical officers, *MDs* medical doctors, *COs* clinical officers, *NOs* nurse officers, *ANOs* assistant nurse officers, *RNs* registered nurses, *VE* vacuum extraction

**Fig. 1:**
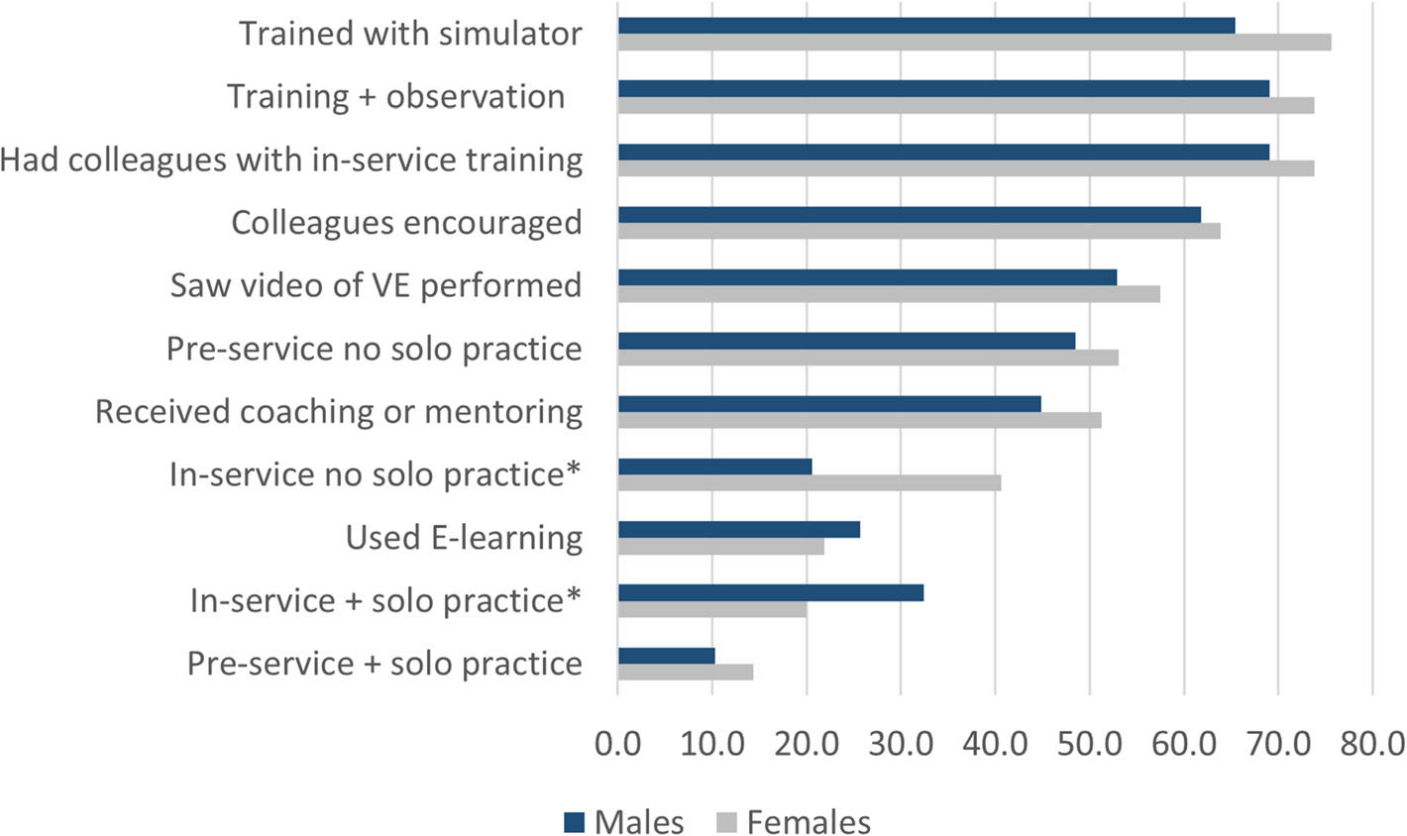
Percentage of providers exposed to training modalities by sex of provider (*p <0.01)

In addition to questions regarding pre- and in-service training in vacuum extraction, providers reported on other training modalities (Table 3). Most of the training elements were significantly associated with recent vacuum extraction performance, especially hands-on practice during in-service training. Two-thirds of the providers with this hands-on experience reported conducting a recent vacuum extraction. More than half (54%) of providers who reported they had used the E-learning module on vacuum extraction developed by Thamini Uhai also reported performing a recent vacuum delivery. About half of providers who received coaching or mentoring from their trainers or supervisors as well as those who reported pre-service hands-on solo practice performed vacuum extraction recently. Two training aspects were not associated with recent performance: pre-service training (27%) and in-service training (31%) *without* the solo hands-on training experience (Fig. 1).

**Table 3:** Proportion of providers who conducted VE in last 3 months, by exposure to training modality

	Conducted VE in last 3 months		
	*n*	%	*p*-value
Total	297	30.6	
In-service training with hands-on solo practice	76	65.8	< 0.001
Used E-learning on VE	70	54.3	< 0.001
Received coaching or mentoring in VE	143	51.0	< 0.001
Pre-service training with hands-on solo practice	38	50.0	0.006
Colleagues encouraged you to conduct VE	186	44.1	< 0.001
Saw a video on how to perform VE	165	41.8	< 0.001
Had colleagues who also had VE in-service training	212	41.0	< 0.001
Had training using simulator or mannequin	211	38.9	< 0.001
Had training with observation of VE	213	38.5	< 0.001
In-service training without solo practice	94	30.9	0.957
Pre-service training without solo practice	151	27.2	0.185
**Categorical scoring summary**			
0–6	19	12.3	< 0.001
7–9	72	50.7	

*VE* vacuum extraction

After conversion of the 11 training opportunities into a continuous score, providers ranged from reporting zero opportunities to 9. We categorized the scores into approximate halves: 0–6 training opportunities vs. 7–9. Only 12% of the first group reported recent performance compared to 51% in the latter group.

Because recent performance of a vacuum delivery varied by sex of the provider (Table 2), we looked at the exposure to these training modalities by sex. Only 2 items were statistically significant, both of which might have contributed to lower performance by female providers. First, a third of men were exposed to solo practice during in-service training (32%) whereas only 20% of females were exposed to solo practice (*p* = 0.015). Second, women disproportionately participated in in-service training that failed to include hands-on experience (41% of women versus 21% among men, *p* < 0.001).

A binary logistic regression model (Table 4) was built to predict recent vacuum extraction performance with the explanatory variables described above in Table 2 that reached statistical significance: administrative district of the workplace, facility type, provider’s sex, training index, and knowledge score. After controlling for the explanatory variables, we found that providers who had been exposed to 7–9 training opportunities compared to providers with less exposure were almost 8 times more likely to have conducted vacuum extraction in the last 3 months. In addition, those who worked in health centers were twice as likely to have conducted a vacuum delivery compared to their hospital-based counterparts, and male providers were also twice as likely as female providers to have recently conducted vacuum delivery. Finally, administrative district also had an independent effect on recent practice of vacuum extraction; providers working in councils other than Kigoma Municipality were 2.7 times as likely to have conducted a vacuum assisted birth compared to their colleagues in Kigoma Municipality. Greater knowledge appeared to increase the likelihood of recent performance of vacuum extraction but failed to reach statistical significance.

**Table 4:** Logistic regression model of performance of vacuum extraction in the last 3 months

	Adjusted Odds Ratios	95% Confidence Intervals	
		Lower	Upper
**Administrative Council**			
Kigoma Municipality	ref.		
Other councils	**2.671**	1.023	6.976
**Type of facility**			
Hospital	ref.		
Health center	**2.107**	1.153	3.850
**Sex of provider**			
Male	**1.951**	1.072	3.550
Female	ref.		
**Training index**			
0–6 modalities	ref.		
7–9 modalities	**7.781**	4.169	14.524
**Knowledge summary score**			
Top 25%	1.822	0.982	3.382
Lower 75%	ref.		

*ref.* reference group; number of cases = 296

Bolded AORs are significant (95% CIs do not contain integer 1)

## Discussion

Vacuum-assisted vaginal delivery is not used widely in sub-Saharan Africa [4, 8, 9, 20]. Despite measured success to re-introduce the practice in Kigoma [14], we knew that uptake of vacuum extraction was highly variable and we knew little about how providers perceived the procedure. This paper explored factors – individual, facility-based and contextual – that potentially influenced the practice of vacuum extraction in the last 3 months. Several factors stood out as more influential than others such as the reporting of having been exposed to different types of training, type of facility in which the provider worked and where that facility was located, and health worker’s sex.

Much has been learned about the art and science of training health workers, for example, we have observed that centralized training is expensive and disruptive to the delivery of services because of absent health workers [21, 22]. Also, didactic trainings are less effective than practice-based learning [21]. The training modality that stood out above others in our study was the experience of hands-on practice of vacuum-assisted birth during in-service training. Two-thirds of those who reported in-service solo experience and half of those with pre-service solo practice had recently performed a vacuum delivery. Our findings confirm what researchers found in 3 regions in Senegal: 64% of the providers interviewed were trained to perform vacuum but only 30% did so as routine practice. The authors attributed this low level to the lack of or limited hands-on practice during training and limited knowledge of the indications, contra-indications and complications of vacuum extraction [20]. Another recent study, this one conducted at the Muhimbili National Hospital in Tanzania, the country’s largest teaching hospital, described an environment of minimal vacuum extraction practice, extremely high cesarean delivery rates, poor knowledge about vacuum extraction, reports of dysfunctional equipment and the need for updated clinical guidelines. The authors concluded that the primary solution would be to support a well-structured training program with hands-on practice for residents and midwives that would help counter a climate of providers’ fear of being blamed for unfavorable outcomes [23].

Practice-based learning in the field of emergency obstetric care is challenged by the rarity of some obstetric emergencies. This was addressed in Kigoma by ensuring that the majority of trainees had access to the use of simulation or mannequins to supplement their learning, and these are known to be effective training aids. A systematic review of operative vaginal delivery demonstrated that vacuum-assisted simulation training decreased many perineal lacerations and newborn injuries, and increased residents’ knowledge regarding vacuum-assisted delivery as well as their comfort performing the procedure immediately following the simulation training, at 4 and at 12-months post-training [24].

One training modality that has been reported to significantly influence the uptake and performance of other maternal and neonatal life-saving procedures is the use of low-dose high-frequency skills-building sessions. Studies in Ghana, Tanzania and Uganda report improved knowledge and skills of providers and better maternal and perinatal outcomes when providers were trained through simulation, case-based learning and small content packages spread over short time intervals [25–28].

Multifaceted and frequent interventions tend to be more successful than one-off interventions [22]. We also found this to be true; as the exposure to different types of training increased, so did the likelihood that the health worker had performed a recent vacuum extraction. Furthermore, the ability of an individual provider to seek additional training on their own (like with E-learning) was also associated with greater performance [22]. This aligns with our finding of a strong correlation between recent vacuum extraction and experience with the Thamini Uhai E-learning platform.

The program’s primary focus was to support rural health centers to provide comprehensive EmONC, which meant that most supported facilities were in districts other than Kigoma Municipality. The fact that those facilities had more (consistent) interventions over time may be one reason that recent performance of vacuum delivery was higher in those administrative districts (personal communication with authors SD and MK). Another possible reason for this finding could relate to the high turnover and internal rotation of maternity ward staff to other departments at Maweni Regional Referral Hospital, located in Kigoma Municipality. Staff from Maweni who were trained in vacuum extraction may not have remained long enough in maternity units for the practice to be sustained. Health centers have fewer staff with less frequent turnover and that may have contributed to higher performance at that level facility (personal communication SD and MK).

We found that recent performance of a vacuum-assisted birth was twice as likely among male providers as female providers but the reasons for this differential are not entirely clear. The decentralization of the EmONC program depended heavily on task-sharing, which meant extending training of vacuum extraction to nurse officers, assistant nurse officers, registered and enrolled nurses. However, the inclusion of these cadres was new and occurred mostly in the later years of programmatic support. They made up 44% of the health workers interviewed for this study and almost two-thirds were women. Assistant medical officers, largely male, make up the professional category most likely to have recently conducted a vacuum delivery. However, the bivariate relationship between professional category and recent practice showed no association as determined by the Chi-square test (*p* = 0.130).

Female disadvantage may be a function of gender dynamics in society and the health system resulting in less confidence or assertiveness in the training arena. In a study on health provider confidence in obstetric clinical skills conducted in Uganda and Zambia, researchers found that being female was associated with lower confidence compared to males after adjusting for cadre, age, and other covariates [29]. Another study conducted with health providers in Nigeria also found that females were less confident than males in their clinical skills to provide obstetric care [30]. However, neither study looked at relationships between confidence, training modalities and actual performance.

Based on this study’s findings, future pre-service and in-service training in vacuum extraction must combine various approaches that allow for frequent hands-on practice and self-directed learning ensuring health providers are knowledgeable, skilled and confident. This could include the development of a package that includes low-dose high-frequency training sessions with simulation and mannequins that are reinforced by accessible self-directed courses (e.g., e-learning modules), and backed up with regular supportive supervision, mentorship and routine clinical audits. To ensure more women are empowered and more confident to take advantage of hands-on solo practice during training, trainers should develop facilitation strategies that address gender dynamics in their training sessions. Ultimately, gender-integrated training should positively impact the quality of decentralized life-saving services.

There were several imitations to this study. The study’s sample was not representative but purposefully selected to learn more about the knowledge, practice and attitudes toward vacuum extraction in an environment where vacuum extraction had been widely introduced, with extensive access to resources (equipment, training, and on-site supportive supervision). Had resources for this study not been an issue, a more complex study design would have allowed us to compare providers who worked in supported facilities with providers working in facilities that received no external support (and where vacuum extraction is rarely used). This might have led us to a different set of findings. Another limitation of our study arose during analysis because we were unable to pull out facility managers and district health officials from the overall sample to see if they differed in knowledge, practice or attitudes.

## Conclusions

To ensure health facilities offer the full range of EmONC services needed to reduce maternal and perinatal mortality, including infrequently provided services such as vacuum extraction, health providers must receive the most effective forms of training. As the number of institutional deliveries and facilities’ capacity to provide cesarean sections increase, it is critical that health providers have a safe alternative to limit cesarean deliveries (indications being equal). Clear actions can be taken to increase and sustain performance of this procedure. By re-designing training curriculum on vacuum extraction for pre-service and in-service training to include structured low-dose high-frequency hands-on practice along with sensitivity to gender dynamics during training sessions, regular supervision and mentorship, and by providing opportunities for self-directed learning (e.g., e-learning modules), the practice of vacuum extraction is likely to increase.

## Supplementary Information

**Additional file 1.** *Knowledge, Attitudes and Practice (KAP) survey questionnaire for providers’ interviews on vacuum assisted birth in 15 public facilities in Kigoma, Tanzania.

## Data Availability

The datasets generated and analyzed for this study are available from the Thamini Uhai Clinical Director (the corresponding author) on reasonable request.

## Abbreviations

AMO: Assistant medical officer
ANO: Assistant nurse officer
CO: Clinical officer
EmONC: Emergency obstetric and newborn care
MD: Medical doctor
NO: Nurse officer
R/CHMT: Regional/District or town council health management team
RN: Registered nurse
VE: Vacuum extraction
