# Supplementary material for: Factors related to the practice of vacuum-assisted birth: findings from provider interviews in Kigoma, Tanzania
Source: BMC Pregnancy Childbirth. 2021 Apr 14;21:302. doi: 10.1186/s12884-021-03738-0 (PMC8048302; doi:10.1186/s12884-021-03738-0)
Supplement: Supplementary file 1 — Additional file 1. *Knowledge, Attitudes and Practice (KAP) survey questionnaire for providers’ interviews on vacuum assisted birth in 15 public facilities in Kigoma, Tanzania. [file 12884_2021_3738_MOESM1_ESM.doc]

**Providers’ interviews on vacuum assisted birth in 15 public facilities in Kigoma, Tanzania**

**Knowledge, Altitude and Practice (KAP) Survey questionnaire**

| **PROVIDER TRAINING, EXPERIENCE AND KAP ON VACUUM EXTRACTION** | | | |
| --- | --- | --- | --- |
|  | Date of Interview: | A. DAY…………………………………………………………..……. [ __|__ ]  B. MONTH………………………………………………………..…. [ __|__]  C. YEAR………………………………………………..…. [ __|__|__|__ ] |  |
|  | Interviewer Code: | ……………………………………………………….……….. |__|__ ] |  |
|  | Facility Name: | _________________________________________________ |  |
|  | Facility Code: | …………………………………………………………….….. [ __|__|__|__ ] |  |
|  | Facility Type: | HOSPITAL…………………………………………………………………………..1  HEALTH CENTER…………………………………………………………….….2 |  |
|  | District Council | BUHIGWE…………………………………………………………………………..1  KABONDO…………………………………………………..……………………..2  KAKONKO…………………………………………………………………………..3  KASULU TC………………………………………………………………………...4  KASULU DC…………………………………………………………………………5  KIGOMA DC……………………………………………………………..………..6  KIGOMA UJIJI MC…………………………….……………………..…………7  UVINZA ……………………………………………………………………………..8 |  |
|  | Result Code: | COMPLETED………………………………………………………………………1  INCOMPLETE……………………………………………………………………..2  POSTPONED (SPECIFY DATE) _______________________3  REFUSED…………………………….………………………………………………4  OTHER (SPECIFY) _____________________________.........5 |  |
|  | Date of data Entry: | DAY………………………………………………………………..……. [ __|__ ]  MONTH……………………………………………………………..…. [ __|__]  YEAR……………………………………………………..…. [ __|__|__|__ ] |  |
|  | Data Entry Clerk Code: | ……………………………………………………………………..……... [ __|__] |  |

| **PARTICIPANT ELIGIBILITY / SCREENING QUESTIONS** | | | |
| --- | --- | --- | --- |
| **Num-ber** | **Question** | **Response Options** | **Skip** |
| E1 | How old were you on your last birthday? | AGE IN YEARS………………………………………….[ __|__ ]  DON’T KNOW / REMEMBER………………….……………88 | END IF LESS THAN 18 YEARS |
| E2 | What is your current occupational category or qualification (or cadre)?  ***ONLY READ THE FOLLOWING IF PROVIDER DOES NOT IMMEDIATELY RESPOND TO THE QUESTION***  *For example are you a registered nurse, midwife, or generalist medical doctor?* | OBSTETRICIAN (OB)…………………………………………….1  GENERAL PRACTITIONER (MD).,………………………….2  ASSISTANT MEDICAL OFFICER (AMO)…………………3  CLINICAL OFFICER (CO)………………………………………..4  NURSE OFFICER / ASSISTANT NURSE OFFICER / REGISTERED NURSE …………………………..…..…………...5  ENROLLED NURSE…………………………………………….….6 |  |

| **SECTION 1. DEMOGRPAHIC AND WORK INFORMATION** | | | | | | | | | | | | |  | |
| --- | --- | --- | --- | --- | --- | --- | --- | --- | --- | --- | --- | --- | --- | --- |
| **N/o** | **Question** | | | | **Response Options** | | |  | | | | |  | |
| 1.1 | Which year did you graduate or complete this qualification? | | | | GRADUATION YEAR  [___] [___] [___] [___] | | |  | | | | |  | |
|  |  | | | | DON’T KNOW YEAR / DON’T REMEMBER | | | 88 | | | | |  | |
| 1.2 | In which month and year did you start working at this facility as a **[insert cadre from QE2?** | | | | CADRE……………………………………..  A. MONTH………………[ __| [ __] | | |  | | | | |  | |
|  |  | | | | B. YEAR……….[ __|_ _|_ _|_ _ ] | | |  | | | | |  | |
|  |  | | | | DON’T KNOW / REMEMBER MONTH  DON’T KNOW / REMEMBER YEAR | | | 88 | | | | |  | |
| 1.3 | Sex | | | | MALE | | | 1 | | | | |  | |
|  |  | | | | FEMALE | | | 2 | | | | |  | |
| **SECTION 2. TRAINING IN VACUUM EXTRACTION** | | | | | | | | | | | | |  | |
| 2.1 | | | Have you ever received any pre-service training to conduct vacuum extraction? | | NO | | | | | | 1 🡪 | | SKIP TO QN 2.8 | |
|  | | |  | | YES | | | | | | 2 | |  | |
| 2.2 | | | Did the pre-service training include hands-on practice with a simulator or mannequin? | | NO | | | | | | 1 | |  | |
|  | | |  | | YES | | | | | | 2 | |  | |
|  | | |  | | DON’T REMEMBER | | | | | | 88 | |  | |
| 2.3 | | | Before the end of the training, did you observe a woman deliver by vacuum extraction? | | NO | | | | | | 1 | |  | |
|  | | |  | | YES | | | | | | 2 | |  | |
|  | | |  | | DON’T REMEMBER | | | | | | 88 | |  | |
| 2.4 | | | Before the end of the training, was it a requirement for you to conduct a vacuum delivery by yourself? | | NO | | | | | | 1 | |  | |
|  | | |  | | YES | | | | | | 2 | |  | |
|  | | |  | | DON’T REMEMBER | | | | | | 88 | |  | |
| 2.5 | | | Before the end of the training, did you perform a vacuum delivery on a woman by yourself? | | NO/DON’T REMEMBER | | | | | | 1 | | SKIP TO QN 2.8 | |
|  | | |  | | YES | | | | | | 2 | |  | |
| 2.6 | | | How many vacuum deliveries did you perform before you qualified (graduated)? | | NUMBER OF VACUUM DELIVERIES PERFORMED [ _ _|_ _] | | | | | |  | |  | |
|  | | |  | | DON’T KNOW / REMEMBER | | | | | | 88 | |  | |
| 2.7 | | | What type of instrument did you use during the pre-service training? | | VACUUM EXTRACTOR WITH SOFT CUP | | | | | | 1 | |  | |
|  | | |  | | VACUUM EXTRACTOR WITH METAL CUP | | | | | | 2 | |  | |
|  | | |  | | KIWI | | | | | | 3 | |  | |
|  | | |  | | DON’T KNOW / REMEMBER | | | | | | 88 | |  | |
| 2.8 | | | Have you ever received any in-service training to conduct vacuum extraction? | | NO | | | | | | 1 | | IF NO PRE-SERVICE **AND** NO IN-SERVICE, SKIP TO SECTION 3  IF YES TO PRE-SERVICE AND NO TO IN-SERVICE, SKIP TO QN 2.20 | |
|  | | |  | | YES | | | | | | 2 | |  | |
| 2.9 | | | Did the in-service training include hands-on practice with a simulator or mannequin? | | NO | | | | | | 1 | |  | |
|  | | |  | | YES | | | | | | 2 | |  | |
|  | | |  | | DON’T REMEMBER | | | | | | 88 | |  | |
| 2.10 | | | Before the end of the training, did you observe a woman have a vacuum delivery? | | NO | | | | | | 1 | |  | |
|  | | |  | | YES | | | | | | 2 | |  | |
|  | | |  | | DON’T REMEMBER | | | | | | 88 | |  | |
| 2.11 | | | Before the end of the training, was it a requirement for you to conduct a vacuum delivery by yourself? | | NO | | | | | | 1 | |  | |
|  | | |  | | YES | | | | | | 2 | |  | |
|  | | |  | | DON’T REMEMBER | | | | | | 88 | |  | |
| 2.12 | | | Before the end of the training, did you perform a vacuum delivery on a woman by yourself? | | NO/DON’T REMEMBER | | | | | | 1 | | IF NO, SKIP TO QN 2.15 | |
|  | | |  | | YES | | | | | | 2 | |  | |
| 2.13 | | | How many vacuum deliveries did you perform before the end of the in-service training program? | | NUMBER OF VACUUM DELIVERIES PERFORMED [ _ _|__ ] | | | | | |  | |  | |
|  | | |  | | DON’T KNOW / REMEMBER | | | | | | 88 | |  | |
| 2.14 | | | What type of instrument did you use during the in-service training? **If more than one instrument was used, circle applicable types)* | | VACUUM EXTRACTOR WITH SOFT CUP | | | | | | 1 | |  | |
|  | | |  | | VACUUM EXTRACTOR WITH METAL CUP | | | | | | 2 | |  | |
|  | | |  | | KIWI | | | | | | 3 | |  | |
|  | | |  | | DON’T KNOW / REMEMBER | | | | | | 88 | |  | |
| 2.15 | | | Was the in-service training you participated in B-EmONC or C-EmONC training where vacuum delivery was just one topic among many topics? | | NO | | | | | | 1 | |  | |
|  | | |  | | YES | | | | | | 2 | |  | |
| 2.16 | | | When was the most recent training that included vacuum delivery? | | WITHIN THE LAST 12 MONTHS | | | | | | 1 | |  | |
|  | | |  | | WITHIN THE LAST 24 MONTHS | | | | | | 2 | |  | |
|  | | |  | | MORE THAN 2 YEARS AGO | | | | | | 3 | |  | |
|  | | |  | | DON’T KNOW / REMEMBER | | | | | | 88 | |  | |
| 2.17 | | | Have you ever participated in a Continuing Medical Education workshop specifically on vacuum delivery? | | NO | | | | | | 1 | |  | |
|  | | |  | | YES | | | | | | 2 | |  | |
| 2.18 | | | Have you participated in more than one in-service training program that included vacuum delivery? | | NO | | | | | | 1 | | SKIP TO QN 2.20 | |
|  | | |  | | YES | | | | | | 2 | |  | |
| 2.19 | | | How many different in-service training programs have you participated in that included vacuum delivery? | | NUMBER OF IN-SERVICE TRAININGS  [ __|__] | | | | | |  | |  | |
|  | | |  | | DON’T KNOW / REMEMBER | | | | | | 88 | |  | |
| 2.20 | | | Since your training(s), have you received any coaching or mentoring from your trainers or supervisors on vacuum delivery? | | NO | | | | | | 1 | |  | |
|  | | |  | | YES | | | | | | 2 | |  | |
| 2.21 | | | Were there other providers from your facility that received in-service training on VE at the same time? | | NO | | | | | | 1 | |  | |
|  | | |  | | YES | | | | | | 2 | |  | |
| 2.22 | | | Is that provider(s) still at your facility? | | NO | | | | | | 1 | | Qn 2.25 | |
|  | | |  | | YES | | | | | | 2 | |  | |
| 2.23 | | | After the training, did your peer worker(s) continue to perform any VE procedures? | | NO | | | | | | 1 | |  | |
|  | | |  | | YES | | | | | | 2 | |  | |
| 2.24 | | | Do your peer workers encourage you to perform VE? | | No | | | | | | 1 | |  | |
|  | | |  | | YES | | | | | | 2 | |  | |
| 2.25 | | | Have you ever used Thamini Uhai (formerly WLF) E-learning modules on VE? | | No | | | | | | 1 | | IF NOT, SKIP TO Qn 2.26 | |
|  | | |  | | YES | | | | | | 2 | |  | |
| 2.26 | | | Did the E-learning modules help you to gain confidence for performing VE procedures? | | NO | | | | | | 1 | |  | |
|  | | |  | | YES | | | | | | 2 | |  | |
|  | | |  | |  | | | | | |  | |  | |
| 2.27 | | | What are the core competencies related to vacuum delivery that you were taught in your pre-service or in-service training session(s)? | | **MENTIONED** | | **DID NOT MENTION** | | | |  | |  | |
|  |  |  | 1. Assessment of maternal and fetal conditions | | 1 | | 0 | | | |  |  |  | |
|  |  |  | 1. Gaining skills to use the vacuum extractor | | 1 | | 0 | | | |  |  |  | |
|  |  |  | 1. Decision-making | | 1 | | 0 | | | |  |  |  | |
|  |  |  | 1. Other (specify) _________________ | | 1 | | 0 | | | |  |  |  | |
| 2.28 | | | Have you ever seen a video on how to perform vacuum delivery? | | NO | | | | | | 1 | |  | |
|  | | |  | | YES | | | | | | 2 | |  | |
| 2.29 | | | Can you name any types of equipment used to assist with a vaginal delivery? | | **MENTIONED** | | **DID NOT MENTION** | | | |  | |  | |
|  |  |  | 1. KIWI | | 1 | | 0 | | | |  |  |  | |
|  |  |  | 1. Mityvac or Mystic | | 1 | | 0 | | | |  |  |  | |
|  |  |  | 1. Malmstorm | | 1 | | 0 | | | |  |  |  | |
|  |  |  | 1. Forceps | | 1 | | 0 | | | |  |  |  | |
|  |  |  | 1. Other (specify) __________ | | 1 | | 0 | | | |  |  |  | |
| **Section 3. Knowledge** | | | | | | | | | | | | |  | |
| 3.1 | | | What are some of the maternal or fetal conditions or indications that would make a woman a good candidate for vacuum delivery? | | **MENTIONED** | | **DID NOT MENTION** | | |  | | |  | |
|  |  |  | 1. Prolonged duration of 2^nd^ stage of labor | | 1 | | 0 | | |  |  |  |  | |
|  |  |  | 1. Maternal exhaustion | | 1 | | 0 | | |  |  |  |  | |
|  |  |  | 1. Need to shorten 2^nd^ stage of labor for maternal medical reason(s) | | 1 | | 0 | | |  |  |  |  | |
|  |  |  | 1. Severe anemia | | 1 | | 0 | | |  |  |  |  | |
|  |  |  | 1. Heart disease / failure | | 1 | | 0 | | |  |  |  |  | |
|  |  |  | 1. Severe pre-eclampsia / eclampsia | | 1 | | 0 | | |  |  |  |  | |
|  |  |  | 1. Suspected or imminent fetal distress (non-reassuring fetal heart rate) in 2^nd^ stage of labor | | 1 | | 0 | | |  |  |  |  | |
|  |  |  | 1. Fetal bradycardia <100/min | | 1 | | 0 | | |  |  |  |  | |
|  |  |  | 1. Fetal tachycardia >160/min | | 1 | | 0 | | |  |  |  |  | |
|  |  |  | 1. Thick meconium stained liquor | | 1 | | 0 | | |  |  |  |  | |
|  |  |  | 1. Other (specify) ________________ | | 1 | | 0 | | |  |  |  |  | |
|  |  |  |  |  |  |  |  |  |  |  |  |  |  |  |
| 3.2 | | | What are some absolute or relative contraindications that would make a woman a poor candidate for vacuum delivery? | | **MENTIONED** | | **DID NOT MENTION** | | |  | | |  | |
|  |  |  | 1. Breech, face or brow presentation, transverse lie | | 1 | | 0 | | |  |  |  |  | |
|  |  |  | 1. Unengaged fetal head: 2/5^th^ or more palpable per abdomen | | 1 | | 0 | | |  |  |  |  | |
|  |  |  | 1. Gestation <34 weeks | | 1 | | 0 | | |  |  |  |  | |
|  |  |  | 1. Incomplete cervical dilation in nulliparous patient | | 1 | | 0 | | |  |  |  |  | |
|  |  |  | 1. Cephalo-pelvic disproportion | | 1 | | 0 | | |  |  |  |  | |
|  |  |  | 1. Incomplete cervical dilation in multiparous patient | | 1 | | 0 | | |  |  |  |  | |
|  | | | 1. Moulding grade 3 (irreducible/ overlapping skull bones | | 1 | | 0 | | |  | | |  | |
|  | | | 1. HIV infection | | 1 | | 0 | | |  | | |  | |
| 3.3 | | | When performing vacuum delivery, what is the maximum number of pulls that should be attempted? | | NUMBER OF PULLS [_ _ _ ] | | | | |  | | |  | |
|  | | |  | | DON’T KNOW / REMEMBER | | | | | 8 | | |  | |
| 3.4 | | | As a caregiver, what do you do if the vacuum cup pops off three times? | | STOP AND PREPARE FOR EMERGENCY C/S | | | | | 1 | | |  | |
|  | | |  | | KEEP TRYING | | | | | 2 | | |  | |
|  | | |  | | DON’T KNOW / REMEMBER | | | | | 8 | | |  | |
| 3.5 | | | The following statements are either true or false; please state which. | | **TRUE** | **FALSE** | | | |  | | |  | |
|  |  |  | 1. An episiotomy should be done with each vacuum delivery. | | 1 | 0 | | | |  |  |  |  | |
|  |  |  | 1. The definition of prolonged 2^nd^ stage of labor is the same for nulliparous and multiparous women. | | 1 | 0 | | | |  |  |  |  | |
|  |  |  | 1. A pudendal block or lidocaine should be given to every woman undergoing a vacuum delivery. | | 1 | 0 | | | |  |  |  |  | |
|  |  |  | 1. The baby’s head should advance with each pull. | | 1 | 0 | | | |  |  |  |  | |
|  |  |  | 1. Each pull should synchronize with the woman’s contractions. | | 1 | 0 | | | |  |  |  |  | |
|  |  |  | 1. A woman who has had more than 4 deliveries is at higher risk of vacuum delivery. | | 1 | 0 | | | |  |  |  |  | |
|  |  |  | 1. Nulliparous women are more likely to benefit from having a vacuum delivery that multiparous women. | | 1 | 0 | | | |  |  |  |  | |
|  |  |  | 1. Women with a previous scar should not have a vacuum delivery. | | 1 | 0 | | | |  |  |  |  | |
|  |  |  | 1. Vacuum delivery can reduce the number of referrals. | | 1 | 0 | | | |  |  |  |  | |
|  |  |  | 1. Vacuum extraction is considered one of the Basic EmOC signal functions. | | 1 | 0 | | | |  |  |  |  | |
|  |  |  | 1. The time between the decision to conduct a vacuum delivery and birth is usually shorter than if an emergency caesarean section were performed. | | 1 | 0 | | | |  |  |  |  | |
|  |  |  | 1. The caregiver should explain to the woman why a vacuum delivery is recommended and receive her oral consent before proceeding. | | 1 | 0 | | | |  |  |  |  | |
|  |  |  | 1. A vacuum extractor works well for rotating the baby’s position. | | 1 | 0 | | | |  |  |  |  | |
|  |  |  | 1. HIV+ status is an absolute contraindication for vacuum delivery. | | 1 | 0 | | | |  |  |  |  | |
|  |  |  | 1. Prophylactic antibiotics after a vacuum delivery is mandatory | | 1 | 0 | | | |  |  |  |  | |
| **Section 4: Experience with vacuum delivery** | | | | | | | | | | | | |  | |
| 4.1 | | | When was the last time you performed a vacuum delivery? | | HAS NEVER PERFORMED A VACUUM DELIVERY (OR NOT SINCE TRAINING) | | | | | 1 | | | 🡪  SKIP TO SECTION 5 | |
|  | | |  | | IN THE LAST 2 WEEKS (THIS WEEK AND LAST WEEK) | | | | 2 | | | |  | |
|  | | |  | | LAST MONTH | | | | 3 | | | |  | |
|  | | |  | | WITHIN THE LAST 3 MONTHS | | | | 4 | | | |  | |
|  | | |  | | DON’T KNOW / REMEMBER | | | | 88 | | | |  | |
| 4.2 | | | In the past 2 weeks, how many times did you perform a vacuum delivery? | | NUMBER OF VD CASES [_ _] [_ _] | | | |  | | | |  | |
|  | | |  | | IF NONE | | | | 00 | | | |  | |
|  | | |  | | DON’T KNOW / REMEMBER | | | | 88 | | | |  | |
| 4.3 | | | In the past 3 months, how many times did you perform a vacuum delivery? | | NUMBER OF VD CASES [_ _][_ _] | | | |  | | | |  | |
|  | | |  | | IF NONE = | | | | 00 | | | | SKIP TO Qn 4.6 | |
|  | | |  | | DON’T KNOW / REMEMBER | | | | 88 | | | | SKIP TO Qn 4.6 | |
| 4.4 | | | Of those in the past 3 months, how many were successful, i.e. the woman had a vaginal birth? | | NUMBER OF SUCCESSFUL VAGINAL BIRTHS [ ___] [____] | | | |  | | | |  | |
|  | | |  | | IF NONE | | | | 00 | | | | SKIP TO Qn 4.6 | |
|  | | |  | | DON’T KNOW / REMEMBER | | | | 88 | | | | SKIP TO Qn 4.6 | |
| 4.5 | | | How many women went on to have an emergency caesarean section? | | NUMBER THAT HAD C/S  [__ _] [____] | | | |  | | | |  | |
|  | | |  | | NONE | | | | 00 | | | |  | |
|  | | |  | | DON’T KNOW / REMEMBER | | | | 88 | | | |  | |
| 4.6 | | | Would you say that over the past 12 months you are performing more vacuum deliveries, fewer vacuum deliveries, or the number has been stable? | | MORE VACUUM DELIVERIES | | | | 1 | | | |  | |
|  | | |  | | FEWER VACUUM DELIVERIES | | | | 2 | | | | 🡪SKIP TO Qn 4.8 | |
|  | | |  | | ABOUT THE SAME NUMBER | | | | 3 | | | | 🡪SKIP TO Qn 4.9 | |
| 4.7 | | | Why do you think you are performing more vacuum deliveries?   |  | **Reasons** | **Mentioned** | **Not mentioned** | | --- | --- | --- | --- | | A | Greater availability of VE equipment | 1 | 0 | | B | Positive support/encouragement from peer workers | 1 | 0 | | C | Positive support/motivation from facility managers | 1 | 0 | | D | Just interested/motivated to perform the procedure | 1 | 0 | | E | Previous positive experience, has done several previous successful VEs | 1 | 0 | | F | Adequate skills/competencies | 1 | 0 | | g | Recent trainings | 1 | 0 | | **h** | **Others, specify:**  **……………………………………………………….………………………………………………………………………………………………** | 1 | 0 | | | | | | |  | | | | SKIP TO Qn. 4.9 | |
| 4.8 | | | Why do you think you are performing fewer Vacuum Deliveries than before? | | | | | |  | | | |  | |
|  |  |  | \|  \| **Reasons** \| **Mentioned** \| **Not mention-ed** \| \| --- \| --- \| --- \| --- \| \| A \| Lack of/broken VE equipment \| 1 \| 0 \| \| B \| Lack of support from peer workers \| 1 \| 0 \| \| C \| Lack of confidence \| 1 \| 0 \| \| D \| Lack of support from facility managers \| 1 \| 0 \| \| E \| Not interested/motivated \| 1 \| 0 \| \| F \| Previous negative experience (loss or serious injury to newborn) \| 1 \| 0 \| \| g \| Inadequate skills/competencies \| 1 \| 0 \| \| **h** \| **Others, specify:**  **……………………………………………………………………………………………………………………………………** \|  \|  \| \|  \|  \| | | | | | |  | | | |  | |
| 4.9 | | | Have you ever had a bad experience as a result of using vacuum extraction? | YES, WITH MOTHER | | | | | 1 | | | |  | |
|  | | |  | YES, WITH NEWBORN | | | | | 2 | | | | SKIP TO Qn 4.11 | |
|  | | |  | YES, WITH BOTH MOTHER & NEWBORN | | | | | 3 | | | |  | |
|  | | |  | NEVER HAD A NEGATIVE EXPERIENCE…………… | | | | | 4 | | | | Skip to Qn 4.12 | |
|  | | |  | DON’T KNOW / REMEMBER | | | | | 88 | | | |  |  |
| 4.10 | | | What complications has a mother (you delivered by vacuum) experienced? | **MENTIONED** | | **DID NOT MENTION** | | |  | | | |  | |
|  |  |  | 1. Episiotomy or cervical tear requiring suturing | 1 | | 0 | | |  |  |  |  | IF NO COMPLICATION OF NEWBORN, SKIP TO Qn 4.12 | |
|  |  |  | 1. Anal sphincter rupture | 1 | | 0 | | |  |  |  |  |  |  |
|  |  |  | 1. Lacerations of vaginal walls | 1 | | 0 | | |  |  |  |  |  |  |
|  |  |  | 1. Cervical tear/laceration | 1 | | 0 | | |  |  |  |  |  |  |
|  |  |  | 1. Other (specify) ____________ | 1 | | 0 | | |  |  |  |  |  |  |
| 4.11 | | | What complications has a newborn you delivered by vacuum experienced? | **MENTIONED** | | **DID NOT MENTION** | | |  | | | |  | |
|  |  |  | 1. Cephalo-hematoma | 1 | | 0 | | |  |  |  |  |  | |
|  |  |  | 1. Apgar score at 5 min <7 | 1 | | 0 | | |  |  |  |  |  | |
|  |  |  | 1. Convulsions | 1 | | 0 | | |  |  |  |  |  | |
|  |  |  | 1. Difficulties breathing (requiring oxygen therapy) | 1 | | 0 | | |  |  |  |  |  | |
|  |  |  | 1. Failure to breast feed | 1 | | 0 | | |  |  |  |  |  | |
|  |  |  | 1. Scalp injury | 1 | | 0 | | |  |  |  |  |  | |
|  |  |  | 1. Retinal hemorrhage | 1 | | 0 | | |  |  |  |  |  | |
|  |  |  | 1. Neonatal jaundice | 1 | | 0 | | |  |  |  |  |  | |
|  |  |  | 1. Intracranial injury | 1 | | 0 | | |  |  |  |  |  | |
|  |  |  | 1. Failed delivery | 1 | | 0 | | |  |  |  |  |  | |
|  |  |  | 1. Death | 1 | | 0 | | |  |  |  |  |  | |
|  |  |  | 1. Others (Specify):………………… |  | |  | | |  |  |  |  |  | |
| 4.12 | | | When learning something new, some things are harder than others. Please tell me, was it easy or difficult for you to learn VE?: (READ ALOUD) | | | Very Easy | | | | | | | 1 | |
|  |  |  |  |  |  | Easy | | | | | | | 2 | |
|  |  |  |  |  |  | Difficult | | | | | | | 3 | |
|  |  |  |  |  |  | Very hard/difficult | | | | | | | 4 | |
| 4.13 | | | Do you have a written protocol for using vacuum extraction in this facility? | NO | | | | | 1 | | | |  | |
|  | | |  | YES | | | | | 2 | | | |  | |
|  | | |  | DON’T KNOW / REMEMBER | | | | | 88 | | | |  | |
| **Section 5: Attitudes towards vacuum delivery** | | | | | | | | | | | | |  | |
| 5.0 | | If given the chance to choose between VE and CS, would you say that women would prefer a vacuum delivery or a caesarean section? | | VACUUM DELIVERY | | | | 1 | | | | |  | |
|  | |  | | CAESAREAN SECTION | | | | 2 | | | | | 🡪SKIP TO Qn 5.2 | |
|  | |  | | DON’T KNOW | | | | 88 | | | | | SKIP TO Qn 5.2 | |
| 5.1 | | Why do you think women would prefer vacuum delivery? (DO NOT READ) | | **MENTIONED** | | **NOT MENTIONED** | |  | | | | |  | |
|  |  | 1. Generally, vacuum is quicker, takes a shorter time | | 1 | | 0 | |  | | | | |  | |
|  |  | 1. Less bleeding | | 1 | | 0 | |  | | | | |  | |
|  |  | 1. Less risk of infections | | 1 | | 0 | |  | | | | |  | |
|  |  | 1. Less painful | | 1 | | 0 | |  | | | | |  | |
|  |  | 1. Less expensive | | 1 | | 0 | |  | | | | |  | |
|  |  | 1. Shorter duration of hospital stay | | 1 | | 0 | |  | | | | |  | |
|  |  | 1. Safer for the mother | | 1 | | 0 | |  | | | | |  | |
|  |  | 1. Safer for the baby | | 1 | | 0 | |  | | | | |  | |
|  |  | 1. Others, specify   ………………………………………………………………………………………………………………… | | | | | | | | | | |  | |
| 5.2 | | Why do you think some women would **prefer a caesarean** section? | | **MENTIONED** | | **NOT MENTIONED** | | | | | |  |  | |
|  |  | 1. A woman doesn’t feel pain with the anaesthesia of a caesarean | | 1 | | 0 | | | | | |  |  | |
|  |  | 1. Caesareans are perceived as very safe for the mother | | 1 | | 0 | | | | | |  |  | |
|  | | 1. Vacuum extraction can cause damage to the baby | | 1 | | 0 | | | | | |  |  | |
|  |  | 1. Women don’t know what VE is but they understand what a caesarean section is | | 1 | | 0 | | | | | |  |  | |
|  |  | 1. Others, specify   …………………………… | | 1 | | 0 | | | | | |  |  | |
| 5.3 | | Would you personally recommend VE be performed on you (if the respondent is the woman) or your wife (for male respondents) or any other close relative or friend? | | NO | | | | | | | | 1 |  | |
|  | |  | | YES | | | | | | | | 2 |  | |
|  | |  | | NOT SURE/DON’T KNOW | | | | | | | | 88 |  | |
| 5.4 | | What do you consider to be the biggest barriers to performing vacuum delivery? | | **SPONTAN-EOUSLY MENTIONED** | | **MENTIONED WHEN PROMPTED** | **NOT CONSIDERED A BARRIER** | | | | |  |  |  |
|  |  | 1. Caregivers are fearful of causing harm. | | 1 | | 2 | 3 | | | | |  |  |  |
|  |  | 1. Caregivers don’t have the confidence to perform. | | 1 | | 2 | 3 | | | | |  |  |  |
|  |  | 1. Equipment is lacking or broken. | | 1 | | 2 | 3 | | | | |  |  |  |
|  |  | 1. Equipment is hard to use. | | 1 | | 2 | 3 | | | | |  |  |  |
|  |  | 1. Not enough caregivers have been trained to use vacuum extraction. | | 1 | | 2 | 3 | | | | |  |  |  |
|  |  | 1. Training does not allow enough hands-on practical learning with women. | | 1 | | 2 | 3 | | | | |  |  |  |
|  |  | 1. The trainers did not use a simulator / mannequin. | | 1 | | 2 | 3 | | | | |  |  |  |
|  |  | 1. Training was too short. | | 1 | | 2 | 3 | | | | |  |  |  |
|  |  | 1. Senior staff do not like vacuum delivery. | | 1 | | 2 | 3 | | | | |  |  |  |
|  |  | 1. District/Councils Management does not allow. | | 1 | | 2 | 3 | | | | |  |  |  |
|  |  | 1. Regional Health Management Team (RHMT) does not allow. | | 1 | | 2 | 3 | | | | |  |  |  |
|  |  | 1. My professional association doesn’t allow. | | 1 | | 2 | 3 | | | | |  |  |  |
|  |  | 1. National /Ministry of Health in Tanzania doesn’t allow. | | 1 | | 2 | 3 | | | | |  |  |  |
|  |  | 1. **Others: Specify**   **…………………………………………………………………...** | | 1 | | 2 | 3 | | | | |  |  |  |
|  |  |  |  |  |  |  |  |  |  |  |  |  |  |  |
| 5.5 | | Do you think it is safe to conduct a vacuum delivery in a health center that does not have the capacity to perform a Caesarean section? | | NO | | | | | | | | 1 |  | |
|  | |  | | YES | | | | | | | | 2 |  | |
|  | |  | | NOT SURE / DON’T KNOW | | | | | | | | 8 |  | |
| 5.6 | | We have come to the end of our interview; do you have any question or recommendations to improve further training and practice of VD in Tanzania? | | NO | | | | | | | | 1 | END OF INTERVIEW | |
|  | |  | | YES | | | | | | | | 2 |  | |
| 5.7 | | What are the questions/recommendations?  1……………………………………………………………………………………………………………………………………………  2……………………………………………………………………………………………………………………………………………  3……………………………………………………………………………………………………………………………………………. | | | | | | | | | | |  | |
